# Supplementary material for: Lead-in therapy targeting PD1 and/or LAG3 imposes distinct immune phenotypes in first-line treatment of metastatic melanoma
Source: medRxiv. 2025 Nov 6:2025.11.04.25339499. Preprint. [Version 1] doi: 10.1101/2025.11.04.25339499 (PMC12637783; doi:10.1101/2025.11.04.25339499)
Supplement: Supplement 1 [file media-1.pdf]

Supplementary tables for

Lead-in therapy targeting PD1 and/or LAG3 distinguishes differential impacts upon the immune response in first-line treatment of metastatic melanoma

Lilit Karapetyan[1.9], Anthony R. Cillo[2,3,4], Shuaichao Wang[5], Arivarasan Karunamurthy[6], Ryan C. Massa[1,10], Anjali Rohatgi[1,11], Christopher Deitrick[7], Yana G. Najjar[1,3], Diwakar Davar[1,3], Jason J. Luke[1,3], Cindy Sander[1], Sheryl R. Kunning[2,3], Amy Rose[1], Elizabeth Rush[1], Marion Joy[8], Riyue Bao[1,7], Hong Wang[5], Tullia C. Bruno[2,3,4,13], Dario A.A. Vignali[2,3,4,13], and John M. Kirkwood[1,3,13, 14]

Affiliations

[1]Department of Medicine, Division of Hematology/Oncology, University of Pittsburgh School of Medicine, Pittsburgh, PA, USA

[2]Department of Immunology, University of Pittsburgh, Pittsburgh, PA, USA

[3]Tumor Microenvironment Center, UPMC Hillman Cancer Center, University of Pittsburgh, Pittsburgh, PA, USA

[4]Cancer Immunology and Immunotherapy Program, UPMC Hillman Cancer Center, Pittsburgh, PA, USA

[5]UPMC Hillman Cancer Center Biostatistics Facility, Pittsburgh, PA, USA

[6]Department of Pathology, University of Pittsburgh School of Medicine, Pittsburgh, PA, USA

[7]UPMC Hillman Cancer Center Bioinformatics services, Pittsburgh, PA, USA

[8]Translational pathology imaging laboratory, UPMC Hillman Cancer Center, Pittsburgh, PA, USA

[9]H. Lee Moffitt Cancer Center & Research Institute, Tampa, FL, USA

[10]Abramson Cancer Center, Perelman School of Medicine, University of Pennsylvania, PA, USA

[11]Division of Oncology, Department of Medicine, Washington University School of Medicine, St Louis, MO, United States; Alvin J. Siteman Cancer Center, St Louis, MO, USA

[12]Center for Systems Immunology, Department of Immunology, University of Pittsburgh, Pittsburgh, PA USA

[13]Co-senior authors

[14]Lead contact

Correspondence: Correspondence: Lilit.Karapetyan@moffitt.org (LK), dvignali@pitt.edu (DAAV), tbruno@pitt.edu (TCB), KirkwoodJM@upmc.edu (JMK)

Supplementary table 1. Treatment-related adverse number of events (percent) that occurred in ≥10% of patients and according to lead-in relatlimab (Rela), nivolumab (Nivo), and combination (Combo) therapy.

Adverse Event	Rela, N=14		Nivo, N=15		Combo, N=14		All, N=43	
	Any grade	*Grade 3,4,5*	*Any grade*	*Grade 3,4,5*	*Any grade*	*Grade 3,4,5*	*Any grade*	*Grade 3,4,5*
Any adverse event	14 (100)	13 (92.9)	15 (100)	12 (80)	14 (100)	9 (64.3)	43 (100)	34 (79.1)
Treatment-related adverse event	10 (71.4)	6 (42.9)	13 (86.7)	7 (46.7)	13 (92.9)	2 (14.3)	36 (83.7)	15 (34.9)
Treatment-related adverse event in 10% of patients								
Fatigue	7 (50)	0 (0)	7 (46.7)	1 (6.7)	3 (21.4)	0 (0)	17 (39.5)	1 (2.3)
Hyponatremia	6 (42.9)	1 (7.1)	8 (53.3)	3 (20)	3 (21.4)	0 (0)	17 (39.5)	4 (9.3)
Serum amylase increased	6 (42.9)	3 (21.4)	3 (20)	0 (0)	6 (42.9)	0 (0)	15 (34.9)	3 (7)
Aspartate aminotransferase increased	5 (35.7)	0 (0)	4 (26.7)	1 (6.7)	3 (21.4)	0 (0)	12 (27.9)	1 (2.3)
Alanine aminotransferase increased	5 (35.7)	0 (0)	4 (26.7)	1 (6.7)	2 (14.3)	0 (0)	11 (25.6)	1 (2.3)
Hypothyroidism	3 (21.4)	0 (0)	3 (20)	0 (0)	3 (21.4)	0 (0)	9 (20.9)	0 (0)
Hypophosphatemia	1 (7.1)	0 (0)	4 (26.7)	0 (0)	4 (28.6)	0 (0)	9 (20.9)	0 (0)
Rash maculo-papular	2 (14.3)	0 (0)	3 (20)	0 (0)	4 (28.6)	0 (0)	9 (20.9)	0 (0)
Nausea	3 (21.4)	0 (0)	3 (20)	0 (0)	2 (14.3)	0 (0)	8 (18.6)	0 (0)
Lipase increased	3 (21.4)	2 (14.3)	3 (20)	1 (6.7)	2 (14.3)	1 (7.1)	8 (18.6)	4 (9.3)
Thyroid stimulating hormone increased	3 (21.4)	0 (0)	3 (20)	0 (0)	2 (14.3)	0 (0)	8 (18.6)	0 (0)
Adrenal insufficiency	3 (21.4)	1 (7.1)	2 (13.3)	0 (0)	2 (14.3)	0 (0)	7 (16.3)	1 (2.3)
Alkaline phosphatase increased	1 (7.1)	0 (0)	3 (20)	1 (6.7)	3 (21.4)	0 (0)	7 (16.3)	1 (2.3)
Pruritus	1 (7.1)	1 (7.1)	2 (13.3)	0 (0)	3 (21.4)	0 (0)	6 (14)	1 (2.3)
Neutrophil count decreased	0 (0)	0 (0)	4 (26.7)	0 (0)	1 (7.1)	0 (0)	5 (11.6)	0 (0)

Note: Colitis: 3 patients experienced colitis of any grade; Encephalitis: 1 patient had encephalitis; Myocarditis: 1 patient had myocarditis.

Supplementary Table 2. Multiplex staining antibody panel

Manufacturer	product#	Target	clone	AR	Block	2º HRP	Opal
Biocare	API3209AA	CD4	EP204	9	Akoya	Akoya	690
Cell Signaling	76437S	CD68	D4B96	6	Akoya	Akoya	520
Cell Signaling	12653S	FoxP3	D608R	6	Akoya	Akoya	570
Leica	NC-L-CD20-L26	CD20	L26	6	Akoya	Akoya	620
BioCare	ACI3160A	CD8	C8/144B	6	Akoya	**Leica PowerVision Poly-HRP Anti-Mouse IgG**	480
Abcam	ab212843	SOX10	SOX10/991	6	Akoya	Akoya	780

Supplementary Table 3. Flow cytometry antibodies

	Fluorophore	Antigen	Dilution Factor	Vendor	Cat#	Clone
Surface	APC-Fire810	CD19	50	BL	302272	HIB19
	BV510	CD1c	50	BL	331534	L-161
	BV605	CD62L	100	BD	562719	DREG-56
	BV650	CD163	100	BD	563888	GHI/61
	BV785	CD141	100	BL	344116	M80
	APC	CD138	100	BL	356506	M115
	Spark Blue 550	CD3	100	BL	344852	SK7
	PE-Cy7	CCR7	150	BL	353226	G043H7
	BV480	CD38	150	BD	566137	HIT2
	BV711	CD25	150	BL	302636	BC96
	PerCP-Cy5.5	CD11b	150	BL	301328	ICRF44
	PE-Cy5	CD27	150	TF	15-0279-42	323
	Alexa700	CD11c	150	BL	301648	3.9
	BV750	CD20	150	BD	747062	2H7
	PE-Dazz	CD16	150	BL	302054	3G8
	BUV615	CD103	300	BD	751258	Ber-ACT8
	BUV737	CD14	300	BD	612763	M5E2
	APC-Cy7	CD66B	300	BL	305126	G10F5
	BUV 661	CD56	400	BD	750478	NCAM16.2
	BUV496	CD8	400	BD	612942	RPA-T8
	BV570	CD45RA	600	BL	304132	H1100
	BUV563	CD4	600	BD	741353	RPA-T4
	BUV395	CD45	600	BD	563792	HI30
	AF488	HLADR	800	BL	307620	L243
	PE	CD33	1000	BL	366608	P67.6
ICS	BV421	Ki67	200	BL	350506	Ki-67
	eFluor450	FoxP3	100	TF	48-4776-42	PCH101

Supplementary figures for

Lead-in therapy targeting PD1 and/or LAG3 imposes distinct immune phenotypes in first-line treatment of metastatic melanoma

Lilit Karapetyan[1,9], Anthony R. Cillo[2,3,4], Shuaichao Wang[5], Arivarasan Karunamurthy[6], Ryan C. Massa[1,10], Anjali Rohatgi[1,11], Christopher Deitrick[7], Yana G. Najjar[1,3], Diwakar Davar[1,3], Jason J. Luke[1,3], Cindy Sander[1], Sheryl R. Kunning[2,3], Amy Rose[1], Sarah Bradley[2,3,4], Elizabeth Rush[1], Marion Joy[8], Riyue Bao[1,7], Hong Wang[5], Dario A.A. Vignali[2,3,4,13], Tullia C. Bruno[2,3,4,13], and John M. Kirkwood[1,3,13, 14]

Affiliations

[1]Department of Medicine, Division of Hematology/Oncology, University of Pittsburgh School of Medicine, Pittsburgh, PA, USA

[2]Department of Immunology, University of Pittsburgh, Pittsburgh, PA, USA

[3]Tumor Microenvironment Center, UPMC Hillman Cancer Center, University of Pittsburgh, Pittsburgh, PA, USA

[4]Cancer Immunology and Immunotherapy Program, UPMC Hillman Cancer Center, Pittsburgh, PA, USA

[5]UPMC Hillman Cancer Center Biostatistics Facility, Pittsburgh, PA, USA

[6]Department of Pathology, University of Pittsburgh School of Medicine, Pittsburgh, PA, USA

[7]UPMC Hillman Cancer Center Bioinformatics services, Pittsburgh, PA, USA

[8]Translational pathology imaging laboratory, UPMC Hillman Cancer Center, Pittsburgh, PA, USA

[9]H. Lee Moffitt Cancer Center & Research Institute, Tampa, FL, USA

[10]Abramson Cancer Center, Perelman School of Medicine, University of Pennsylvania, PA, USA

[11]Division of Oncology, Department of Medicine, Washington University School of Medicine, St Louis, MO, United States; Alvin J. Siteman Cancer Center, St Louis, MO, USA

[12]Center for Systems Immunology, Department of Immunology, University of Pittsburgh, Pittsburgh, PA USA

[13]Co-senior authors

[14]Lead contact

Correspondence: Lilit.Karapetyan@moffitt.org (LK), dvignali@pitt.edu (DAAV), tbruno@pitt.edu (TCB), KirkwoodJM@upmc.edu (JMK)

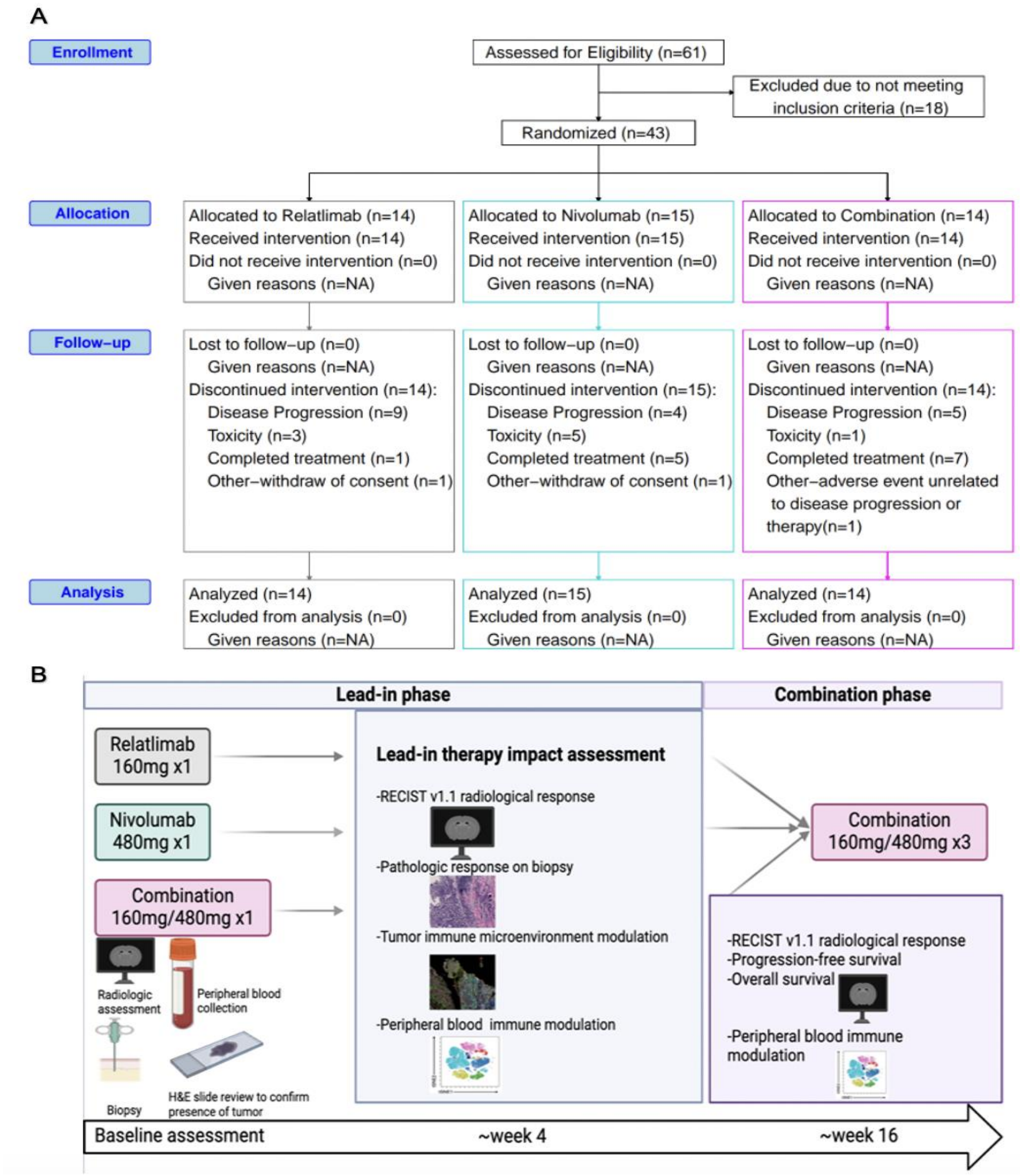

Supplementary figure 1: Clinical trial schema. (A) CONSORT diagram depicting patient screening and disposition. (**B)** Clinical trial schema depicting assessment timepoints for radiological response, pathologic response, tumor immune microenvironment and peripheral blood immunophenotyping changes in lead-in and combination-phases.

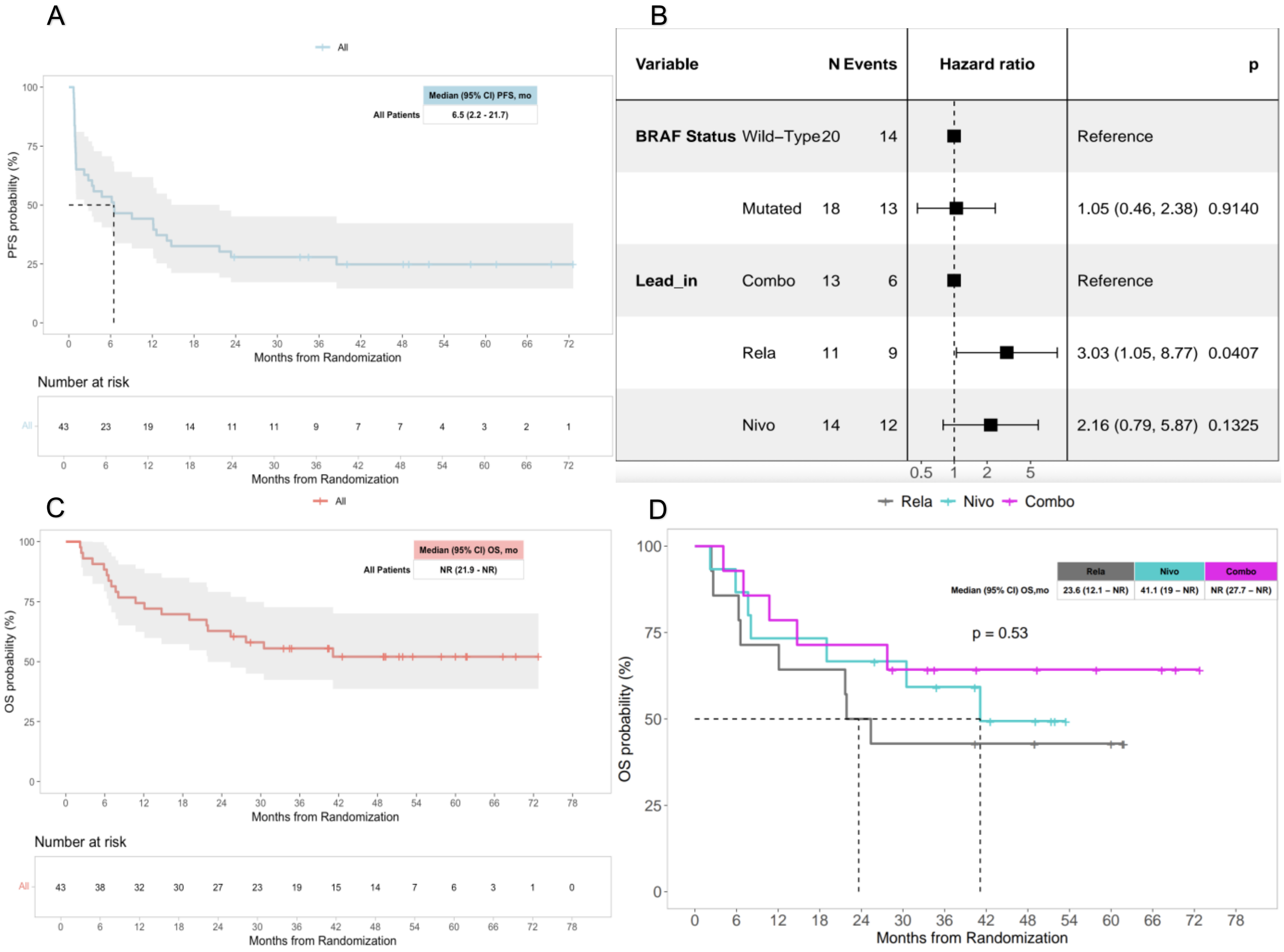

A
All
Median (95% CI) PFS, mo
All Patients
6.5 (2.2 - 21.7)
PFS probability (%)
100
75
50
25
0
0 6 12 18 24 30 36 42 48 54 60 66 72
Months from Randomization
Number at risk
All
43 23 19 14 11 11 9 7 7 4 3 2 1
0 6 12 18 24 30 36 42 48 54 60 66 72
Months from Randomization
B
Variable
N
Events
Hazard ratio
p
BRAF Status
Wild–Type 20 14
Reference
Mutated 18 13
1.05 (0.46, 2.38) 0.9140
Lead_in
Combo 13 6
Reference
Rela 11 9
3.03 (1.05, 8.77) 0.0407
Nivo 14 12
2.16 (0.79, 5.87) 0.1325
0.5 1 2 5
C
All
Median (95% CI) OS, mo
All Patients
NR (21.9 - NR)
OS probability (%)
100
75
50
25
0
0 6 12 18 24 30 36 42 48 54 60 66 72 78
Months from Randomization
Number at risk
All
43 38 32 30 27 23 19 15 14 7 6 3 1 0
0 6 12 18 24 30 36 42 48 54 60 66 72 78
Months from Randomization
D
Rela
Nivo
Combo
Rela
Nivo
Combo
Median (95% CI) OS,mo
23.6 (12.1 – NR)
41.1 (19 – NR)
NR (27.7 – NR)
p = 0.53
OS probability (%)
100
75
50
25
0
0 6 12 18 24 30 36 42 48 54 60 66 72 78
Months from Randomization

Supplementary figure 2: Clinical outcomes (A) Progression-free survival (PFS) among all patients. (**B)** Forest Plot for Cox proportional hazards model showing the impact of lead-in therapy after adjusting for *BRAFv600* mutation status. **(C)** Overall survival (OS) among all patients. (**D)** OS according to lead-in arm.

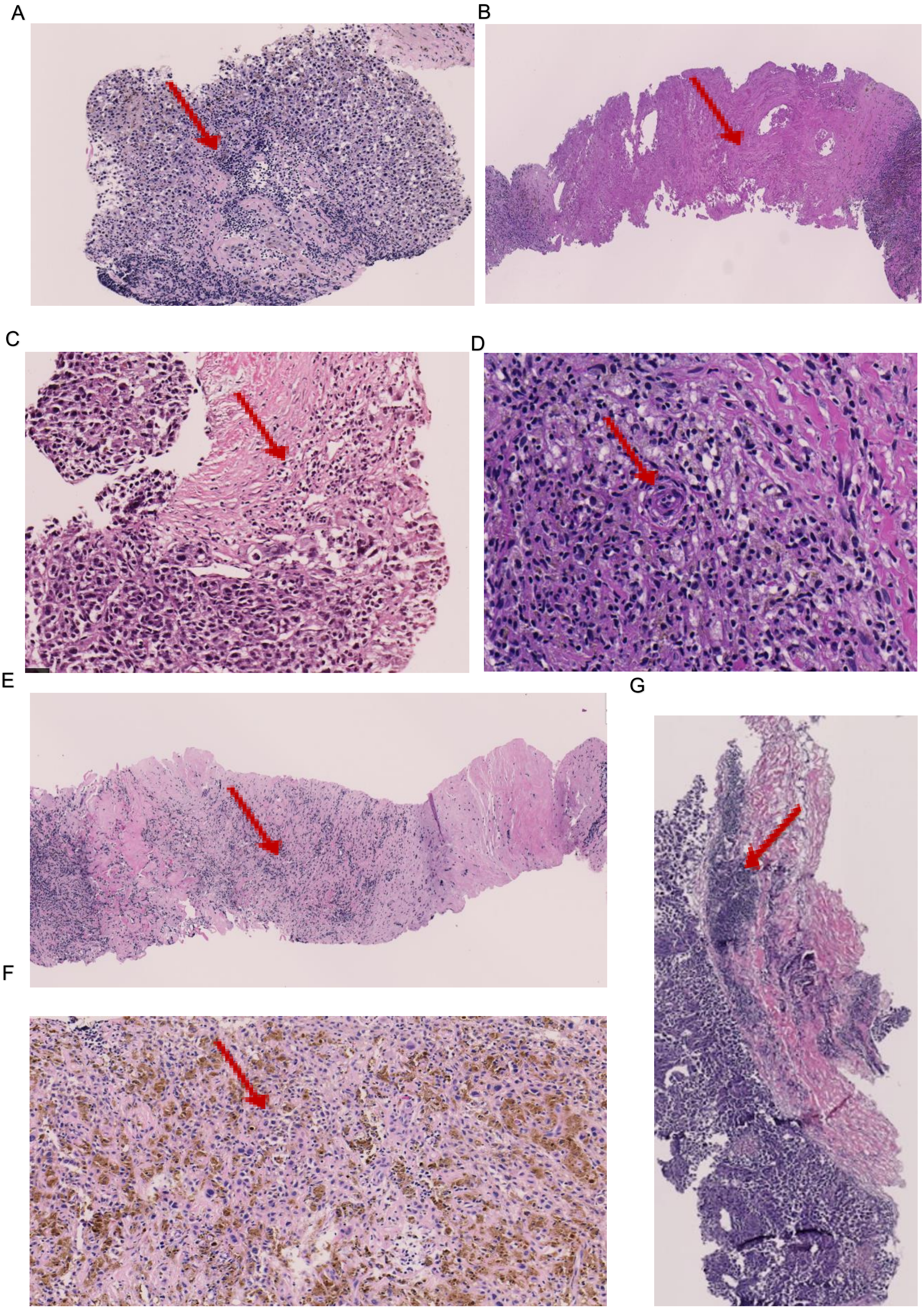
A
B
C
D
E
G
F

Supplementary figure 3: Pathologic response assessment using H&E. (A) tumor-infiltrating lymphocytes. (**B)** absence of viable tumor. (**C)** plasma cells. (**D)** neovascularization. (**E)** fibrosis. (**F)** viable tumor with melanosis. (**G)** lymphoid aggregates.

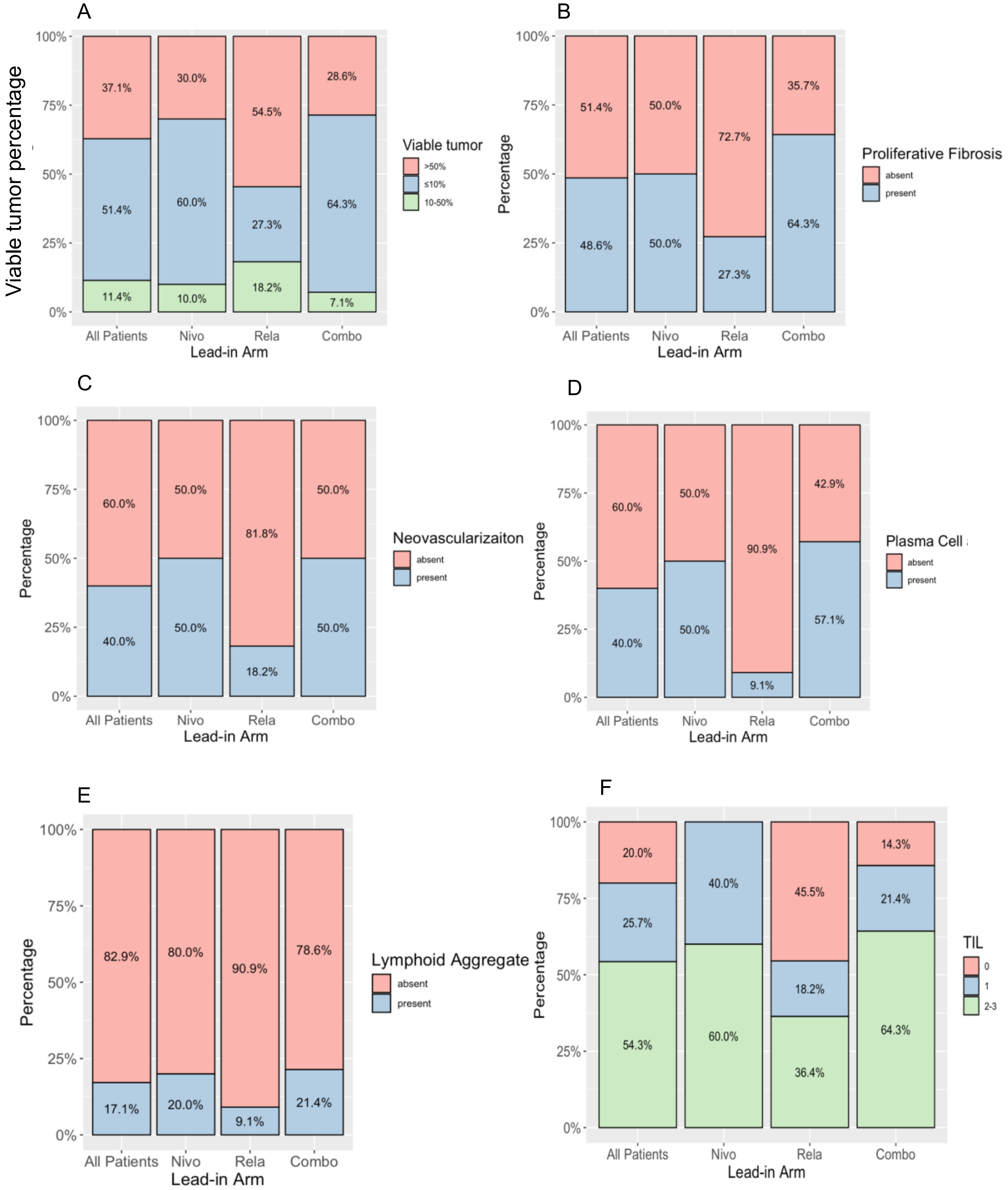
A
Viable tumor percentage
Viable tumor
>50%
≤10%
10-50%
37.1%
30.0%
54.5%
28.6%
51.4%
60.0%
27.3%
64.3%
11.4%
10.0%
18.2%
7.1%
B
Percentage
Proliferative Fibrosis
absent
present
51.4%
50.0%
72.7%
35.7%
48.6%
50.0%
27.3%
64.3%
C
Percentage
Neovascularizaiton
absent
present
60.0%
50.0%
81.8%
50.0%
40.0%
50.0%
18.2%
50.0%
D
Percentage
Plasma Cell
absent
present
60.0%
50.0%
90.9%
42.9%
40.0%
50.0%
9.1%
57.1%
E
Percentage
Lymphoid Aggregate
absent
present
82.9%
80.0%
90.9%
78.6%
17.1%
20.0%
9.1%
21.4%
F
Percentage
TIL
0
1
2-3
20.0%
25.7%
54.3%
40.0%
60.0%
45.5%
18.2%
36.4%
14.3%
21.4%
64.3%
0%
25%
50%
75%
100%
All Patients
Nivo
Rela
Combo
Lead-in Arm

Supplementary figure 4: Pathologic response parameters among 3 lead-in arms. The percentages are depicted among all patients and nivolumab (nivo), relatlimab (rela), and combination (combo) arms, respectively. **(A)** residual viable tumor. (**B)** proliferative fibrosis (present/absent). (**C)** neovascularization (present/absent). (**D)** plasma cells (present/absent). (**E)** lymphoid aggregate (present/absent). (**F)** tumor-infiltrating lymphocytes (0 vs 1 vs 2-3).

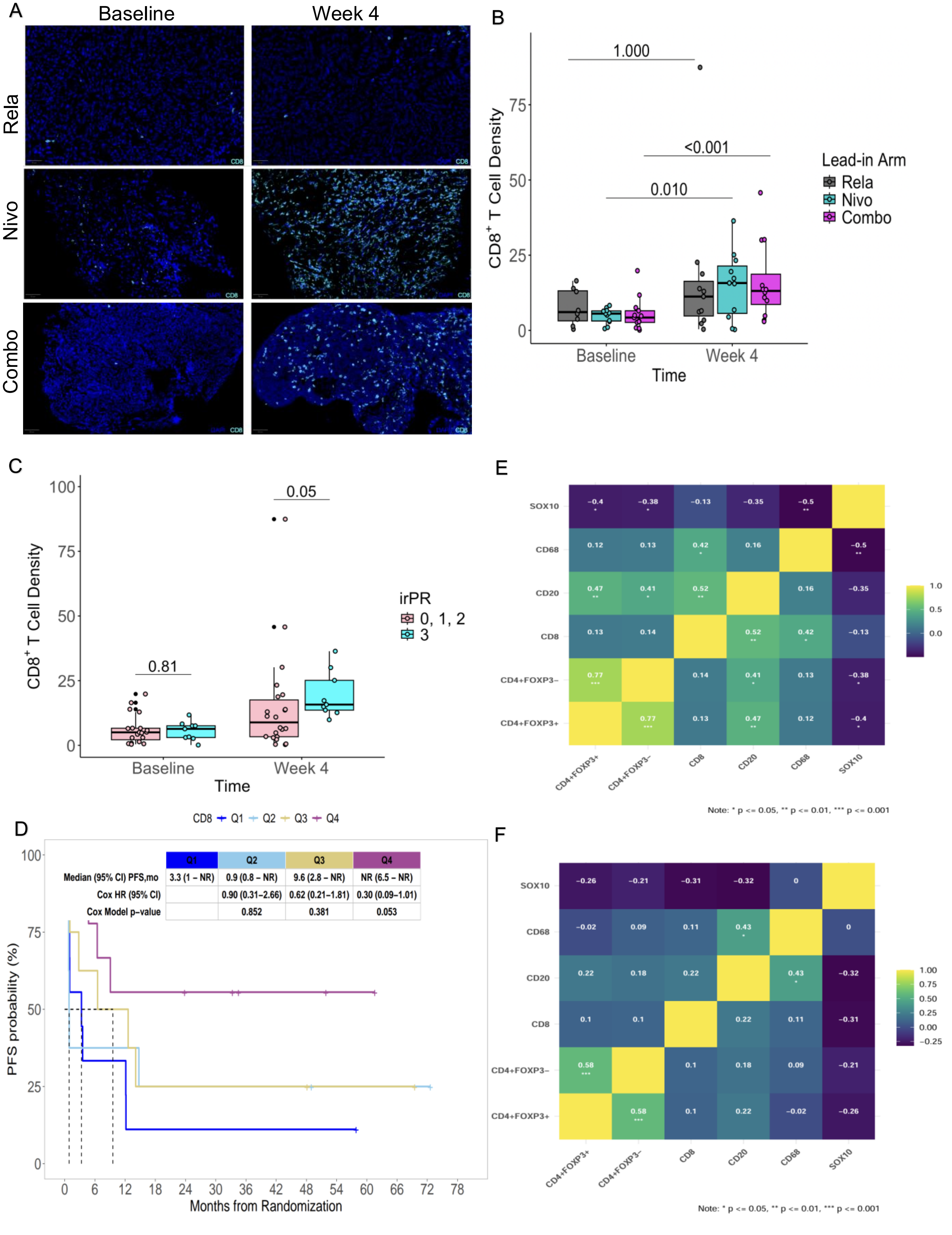
A
Baseline
Week 4
Rela
Nivo
Combo
B
1.000
<0.001
0.010
CD8+ T Cell Density
Baseline
Week 4
Time
Lead-in Arm
Rela
Nivo
Combo
C
0.05
0.81
CD8+ T Cell Density
irPR
0, 1, 2
3
Baseline
Week 4
Time
D
CD8 Q1 Q2 Q3 Q4
Q1 Q2 Q3 Q4
Median (95% CI) PFS,mo 3.3 (1 – NR) 0.9 (0.8 – NR) 9.6 (2.8 – NR) NR (6.5 – NR)
Cox HR (95% CI) 0.90 (0.31–2.66) 0.62 (0.21–1.81) 0.30 (0.09–1.01)
Cox Model p-value 0.852 0.381 0.053
PFS probability (%)
Months from Randomization
E
SOX10
CD68
CD20
CD8
CD4+FOXP3−
CD4+FOXP3+
Note: * p <= 0.05, ** p <= 0.01, *** p <= 0.001
F
SOX10
CD68
CD20
CD8
CD4+FOXP3−
CD4+FOXP3+
Note: * p <= 0.05, ** p <= 0.01, *** p <= 0.001

Supplementary figure 5: Lead-in therapy induced immune changes in tumor microenvironment. (A) Combination (combo) and nivolumab (nivo) but not relatlimab (rela) lead-in therapies led to significant increase in $CD8^+$ T cell density. (**B)** The comparison was performed between baseline and week 4 data. p values were calculated using Wilcoxon signed-rank test. (**C)** $CD8^+$ T cell density at baseline and week 4 in association with immune related pathologic response (irPR). Box plot demonstrates significant increase in week 4 $CD8^+$ T cell density among irPR=3 (major pathologic response on biopsy) vs irPR=0,1,2. (**D)** PFS according to $CD8^+$ T cell density at week 4. (**E)** Correlation plot of cell densities at baseline. (**F)** week 4 samples. Correlation coefficient and p values are depicted in the figure.

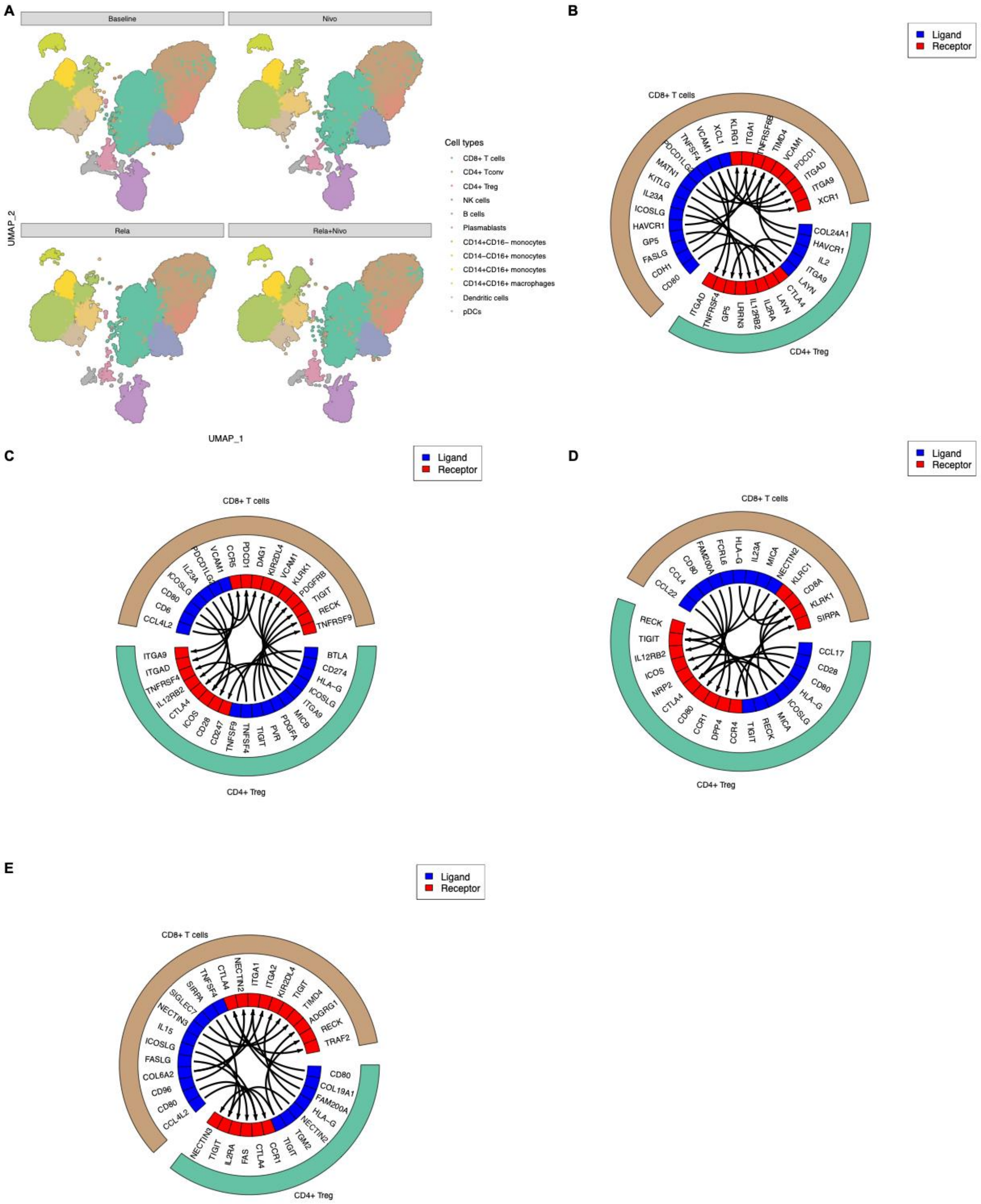
A
Baseline
Nivo
Rela
Rela+Nivo
UMAP_2
UMAP_1
Cell types
CD8+ T cells
CD4+ Tconv
CD4+ Treg
NK cells
B cells
Plasmablasts
CD14+CD16− monocytes
CD14−CD16+ monocytes
CD14+CD16+ monocytes
CD14+CD16+ macrophages
Dendritic cells
pDCs
B
Ligand
Receptor
CD8+ T cells
CD4+ Treg
PDCD1LG2
TNFSF4
VCAM1
XCL1
KLRG1
ITGA1
TNFRSF8
TIMD4
VCAM1
PDCD1
ITGAD
ITGA9
XCR1
MATN1
KITLG
IL23A
ICOSLG
HAVCR1
GP5
FASLG
CDH1
CD80
COL24A1
HAVCR1
IL2
ITGA9
LAYN
LAYN
CTLA4
IL2RA
IL12RB2
LRRN3
GP5
TNFRSF4
ITGAD
C
Ligand
Receptor
CD8+ T cells
CD4+ Treg
PDCD1LG2
VCAM1
CCR5
PDCD1
DAG1
KIR2DL4
VCAM1
KLRK1
PDGFRB
TIGIT
RECK
TNFRSF9
IL23A
ICOSLG
CD80
CD6
CCL4L2
ITGA9
ITGAD
TNFRSF4
IL12RB2
CTLA4
ICOS
CD28
CD247
TNFSF9
TNFSF4
TIGIT
PVR
PDGFA
MICB
ITGA9
ICOSLG
HLA−G
CD274
BTLA
D
Ligand
Receptor
CD8+ T cells
CD4+ Treg
CCL4
CD80
FAM200A
FCRL6
HLA−G
IL23A
MICA
NECTIN2
KLRC1
CD8A
KLRK1
SIRPA
CCL22
RECK
TIGIT
IL12RB2
ICOS
NRP2
CTLA4
CD80
CCR1
DPP4
CCR4
TIGIT
RECK
MICA
ICOSLG
HLA−G
CD80
CD28
CCL17
E
Ligand
Receptor
CD8+ T cells
CD4+ Treg
SIRPA
TNFSF4
CTLA4
NECTIN2
ITGA1
ITGA2
KIR2DL4
TIGIT
TIMD4
ADGRG1
RECK
TRAF2
SIGLEC7
NECTIN3
IL15
ICOSLG
FASLG
COL6A2
CD96
CD80
CCL4L2
CD80
COL19A1
FAM200A
HLA−G
NECTIN2
TGM2
TIGIT
CCR1
CTLA4
FAS
IL2RA
TIGIT
NECTIN3

Supplementary figure 6: CD8$^+$ T cell and FOXP3$^+$ T cell interaction analysis. (**A)** UMAP plot illustrating immune cell subsets identified within tumor immune microenvironment at baseline and week 4 across 3 lead-in arms. (**B)** Circos plot for top ligand-receptor interactions at baseline. (**C)** At week 4 in relatlimab (Rela) lead-in arm. (**D)** Nivolumab (Nivo) lead-in arm. (**E)** Combination (Combo) lead-in arm.

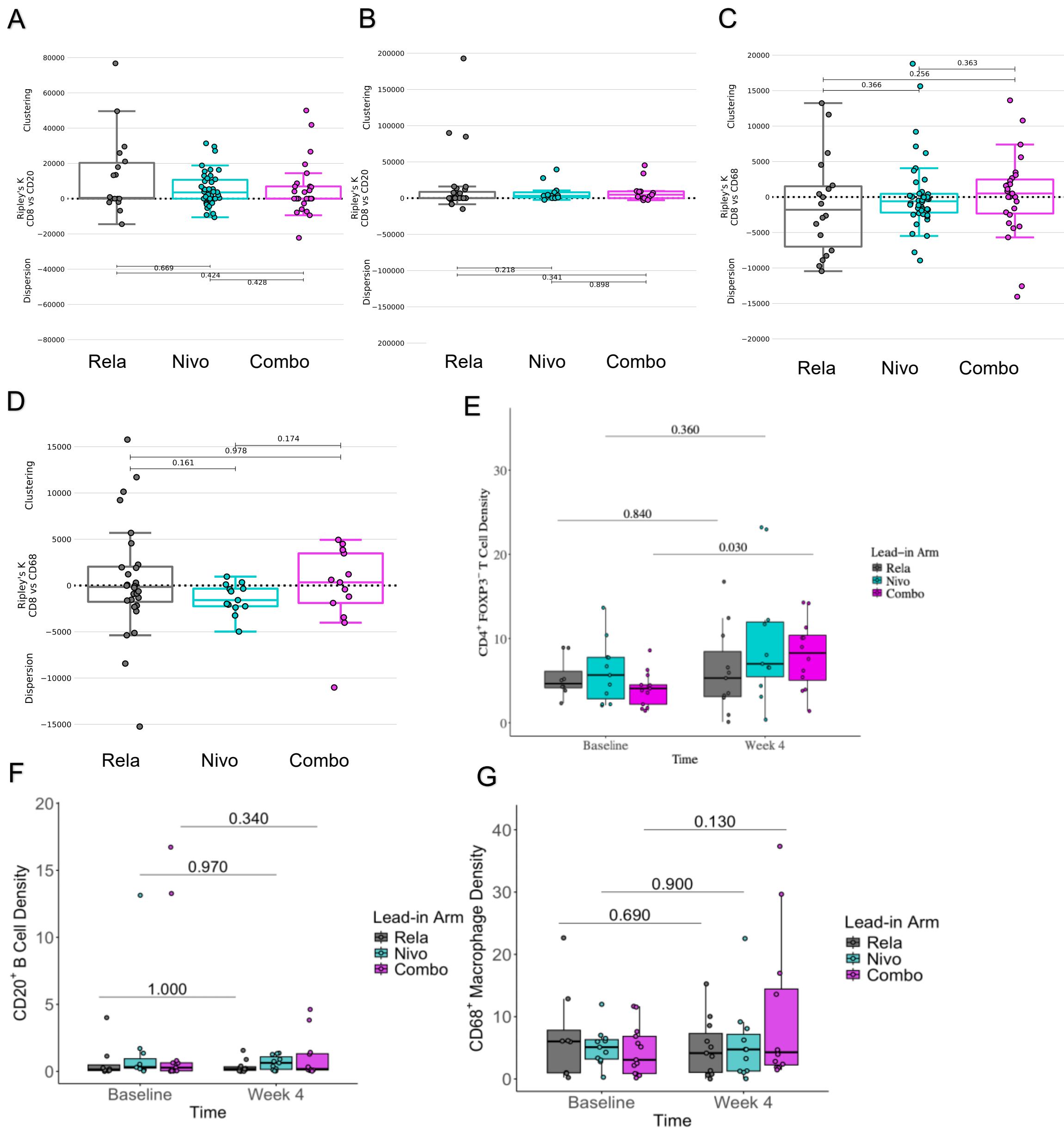
A
Ripley's K
CD8 vs CD20
Clustering
Dispersion
0.669
0.424
0.428
Rela
Nivo
Combo
B
Ripley's K
CD8 vs CD20
Clustering
Dispersion
0.218
0.341
0.898
Rela
Nivo
Combo
C
Ripley's K
CD8 vs CD68
Clustering
Dispersion
0.363
0.256
0.366
Rela
Nivo
Combo
D
Ripley's K
CD8 vs CD68
Clustering
Dispersion
0.174
0.978
0.161
Rela
Nivo
Combo
E
CD4+ FOXP3− T Cell Density
0.360
0.840
0.030
Baseline
Week 4
Time
Lead-in Arm
Rela
Nivo
Combo
F
CD20+ B Cell Density
0.340
0.970
1.000
Baseline
Week 4
Time
Lead-in Arm
Rela
Nivo
Combo
G
CD68+ Macrophage Density
0.130
0.900
0.690
Baseline
Week 4
Time
Lead-in Arm
Rela
Nivo
Combo

Supplementary figure 7: Lead-in therapy induced CD4$^+$ T cell, CD20$^+$ B cell, CD68$^+$ macrophage immune clustering and changes in tumor microenvironment. **(A)** Box plot indicates baseline colocalization patterns of CD8$^+$ T cells and CD20$^+$ B cells. Ripley's K function is illustrated on y axis. Higher numbers indicate clustering and lower numbers indicate dispersion of these two-cell types. The comparison of baseline samples among 3 lead-in arms reveals no significant differences in colocalization patterns. P values were calculated using a Wilcoxon rank-sum test. (**B)** Box plot indicates week 4 colocalization patterns of CD8$^+$ T cells and CD20$^+$ B cells. The comparison of week 4 samples among 3 lead-in arms reveals no significant differences in colocalization patterns. P values were calculated using a Wilcoxon rank-sum test. (**C)** Box plot indicates baseline colocalization patterns of CD8$^+$ T cells and CD68$^+$ macrophages. Ripley's K function is illustrated on y axis. Higher numbers indicate clustering and lower numbers indicate dispersion of these two-cell types. The comparison of baseline samples among 3 lead-in arms reveals no significant differences in colocalization patterns. P values were calculated using a Wilcoxon rank-sum test. (**D)** Box plot indicates week 4 colocalization patterns of CD8$^+$ T cells and CD68$^+$ macrophages. Ripley's K function is illustrated on y axis. Higher numbers indicate clustering and lower numbers indicate dispersion of these two-cell types. The comparison of week 4 samples among 3 lead-in arms reveals no significant differences in colocalization patterns. P values were calculated using a Wilcoxon rank-sum test. (**E)** Combo but not nivo or rela lead-in therapy led to significant increase in CD4$^+$FOXP3$^-$ T cells. p values were calculated using Wilcoxon signed-rank test. (**F)** No significant changes were observed on CD20$^+$ B cell density among 3 lead-in arms. (**G)** No significant changes were observed on CD68$^+$ macrophage density among 3 lead-in arms.

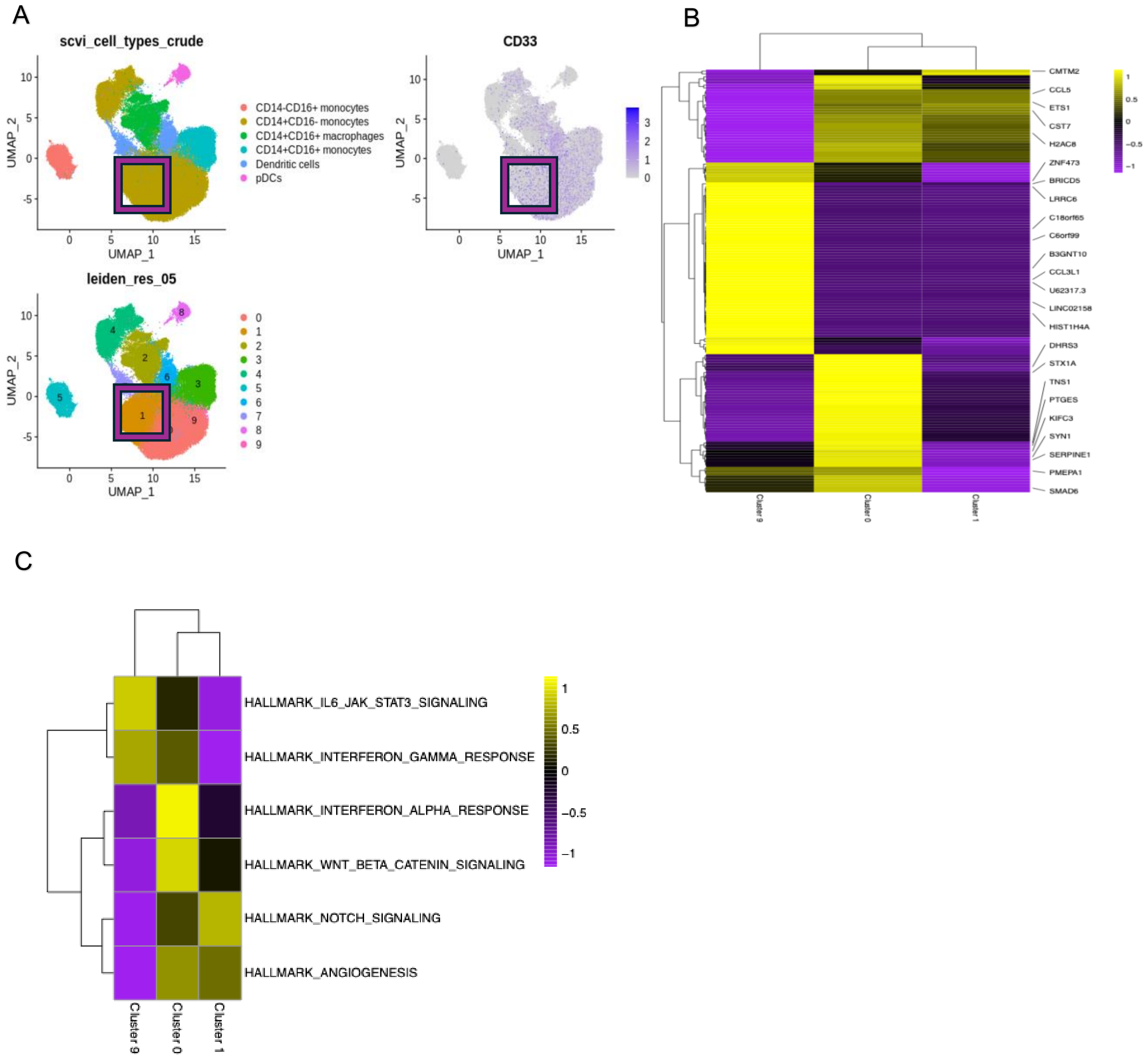

A
scvi_cell_types_crude
CD14-CD16+ monocytes
CD14+CD16- monocytes
CD14+CD16+ macrophages
CD14+CD16+ monocytes
Dendritic cells
pDCs
CD33
leiden_res_05
UMAP_1
UMAP_2
B
CMTM2
CCL5
ETS1
CST7
H2AC8
ZNF473
BRICD5
LRRC6
C18orf65
C6orf99
B3GNT10
CCL3L1
U62317.3
LINC02158
HIST1H4A
DHRS3
STX1A
TNS1
PTGES
KIFC3
SYN1
SERPINE1
PMEPA1
SMAD6
Cluster 9
Cluster 0
Cluster 1
C
HALLMARK_IL6_JAK_STAT3_SIGNALING
HALLMARK_INTERFERON_GAMMA_RESPONSE
HALLMARK_INTERFERON_ALPHA_RESPONSE
HALLMARK_WNT_BETA_CATENIN_SIGNALING
HALLMARK_NOTCH_SIGNALING
HALLMARK_ANGIOGENESIS
Cluster 9
Cluster 0
Cluster 1

Supplementary figure 8: CD33dim classical monocytes demonstrate a unique transcriptional signature. (A) Leiden clustering identified 9 distinct clusters (clusters 0 through 9) of myeloid cells in PBMC. Square highlights CD33 dim classical monocytes (cluster 1). (**B)** Heat map reveals differentially expressed genes across classical monocytes (clusters 0, 1, 9). (**C)** Gene set enrichment analysis across classical monocytes (clusters 0, 1, 9).

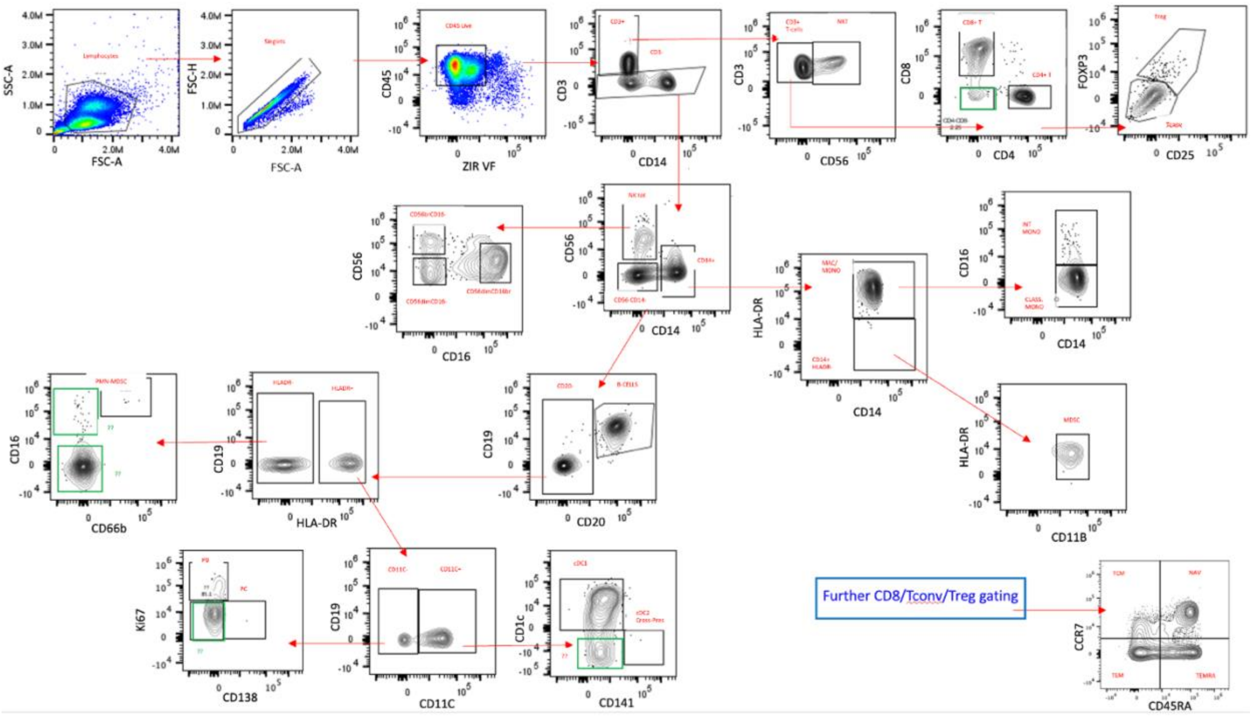

Supplementary figure 9: Gating strategy of basic immune subpopulations.
